# G2/M Checkpoint Abrogation With Selective Inhibitors Results in Increased Chromatid Breaks and Radiosensitization of 82-6 hTERT and RPE Human Cells

**DOI:** 10.3389/fpubh.2021.675095

**Published:** 2021-05-28

**Authors:** Aggeliki Nikolakopoulou, Aashish Soni, Martha Habibi, Pantelis Karaiskos, Gabriel Pantelias, Georgia I. Terzoudi, George Iliakis

**Affiliations:** ^1^Laboratory of Health Physics, Radiobiology and Cytogenetics, Institute of Nuclear and Radiological Sciences and Technology, Energy and Safety, National Centre for Scientific Research “Demokritos”, Athens, Greece; ^2^Medical Physics Laboratory, Medical School, National and Kapodistrian University of Athens, Athens, Greece; ^3^Institute of Medical Radiation Biology, Medical School, University of Duisburg-Essen, Essen, Germany

**Keywords:** chromatid breaks, chromosomal radiosensitivity, G2-M checkpoint, DDR inhibitors, G2-assay

## Abstract

While technological advances in radiation oncology have led to a more precise delivery of radiation dose and a decreased risk of side effects, there is still a need to better understand the mechanisms underlying DNA damage response (DDR) at the DNA and cytogenetic levels, and to overcome tumor resistance. To maintain genomic stability, cells have developed sophisticated signaling pathways enabling cell cycle arrest to facilitate DNA repair via the DDR-related kinases and their downstream targets, so that DNA damage or DNA replication stress induced by genotoxic therapies can be resolved. ATM, ATR, and Chk1 kinases are key mediators in DDR activation and crucial factors in treatment resistance. It is of importance, therefore, as an alternative to the conventional clonogenic assay, to establish a cytogenetic assay enabling reliable and time-efficient results in evaluating the potency of DDR inhibitors for radiosensitization. Toward this goal, the present study aims at the development and optimization of a chromosomal radiosensitivity assay using the DDR and G2-checkpoint inhibitors as a novel modification compared to the classical G2-assay. Also, it aims at investigating the strengths of this assay for rapid radiosensitivity assessments in cultured cells, and potentially, in tumor cells obtained from biopsies. Specifically, exponentially growing RPE and 82-6 hTERT human cells are irradiated during the G2/M-phase transition in the presence or absence of Caffeine, VE-821, and UCN-1 inhibitors of ATM/ATR, ATR, and Chk1, respectively, and the induced chromatid breaks are used to evaluate cell radiosensitivity and their potency for radiosensitization. The increased yield of chromatid breaks in the presence of DDR inhibitors, which underpins radiosensitization, is similar to that observed in cells from highly radiosensitive AT-patients, and is considered here as 100% radiosensitive internal control. The results highlight the potential of our modified G2-assay using VE-821 to evaluate cell radiosensitivity, the efficacy of DDR inhibitors in radiosensitization, and reinforce the concept that ATM, ATR, and Chk1 represent attractive anticancer drug targets in radiation oncology.

## Introduction

Radiation therapy (RT) has become one of the most common treatments for many types of cancer, and while rapid technological advances have led to a more precise delivery of radiation dose and, thus, to a decreased risk of side effects, there is a need to further improve RT by overcoming tumor cell radioresistance (1). Double-stranded breaks (DSBs) are considered the most cytotoxic type of DNA damage being induced by exogenous agents such as ionizing radiation (IR) and chemotherapeutic drugs (2–4). DSBs delay cells from entering mitosis and cause chromosomal aberrations, mitotic cell death, and tumorigenesis (2, 4). To maintain genomic stability after genotoxic treatments, cells activate a complex network of sophisticated signaling pathways, known as the DNA damage response (DDR), which includes the activation of cell cycle checkpoints that slow down or arrest cell cycle progression to facilitate DNA repair or, alternatively, apoptosis (4, 5). During cancer therapy which utilizes agents that induce DNA damage and/or replication stress, DDR activation leads to tumor resistance. The Ataxia telangiectasia mutated kinase (ATM), ataxia telangiectasia-Rad3 related kinase (ATR), as well as the checkpoint kinases 1 (Chk1) and 2 (Chk2) are key components of DDR (6–8). Although a considerable body of information is available regarding the function of these kinases, there is still a need to further elucidate the mechanisms underpinning tumor cell resistance to radiation.

Indeed, decreasing tumor cell resistance by inhibiting these kinases is an attractive therapeutic concept in radiation oncology and cancer therapy (8). The ATM and ATR kinases are important in the activation of checkpoints and play crucial roles in the cellular responses to DNA damage and replication stress; they are considered, therefore, promising targets for radiosensitization (9). Current experimental work is focusing on the hypothesis that the use of potent inhibitors of DDR components can selectively sensitize cancer cells at the molecular level to DNA damaging treatments, and, thus, enhance the efficacy of conventional genotoxic cancer therapies (i.e., radiotherapy and chemotherapy) (10, 11). Indeed, several proof-of-principle studies have demonstrated that the functional loss of ATR leads to the abrogation of the DNA damage-induced G2/M cell cycle arrest and sensitization of cells to IR (9, 12–15).

The earliest inhibitor of phosphatidylinositol 3-kinase (PI3K) related family of protein kinases (PIKK) to be discovered was the fungal metabolite wortmannin (16). Wortmannin showed a very strong potency against almost all PI3K members. However, low selectivity, irreversible inhibition, and the high *in vitro* and *in vivo* toxicity of this compound prevented its further use in cancer therapy (16, 17). The other naturally occurring inhibitor of ATM and ATR is Caffeine (18–20). The attractiveness of ATM and ATR kinases as targets is well-reflected in the intensive efforts of several pharmaceutical companies and academic institutions to develop small selective inhibitors for these kinases (20, 21). Some advanced ATM inhibitors (e.g., KU-60019) exhibit strong and safe radio- and chemosensitization in tumor cells, and suppress cell proliferation and migration (22). Some studies even find radiosensitization using DNA repair pathway inhibitors in various types of cancer without severe toxicity in normal tissue (23–25).

It is of importance, therefore, to establish a cytogenetic assay enabling reliable and time-efficient results in evaluating the potency of DDR inhibitors for radiosensitization. The main objectives of the present study are: (1) To develop and optimize a G2-chromosomal radiosensitivity assay using the DDR and G2-checkpoint inhibitors as a novel modification compared to the classical G2-assay. (2) To investigate the strengths of this assay, as an alternative to the conventional clonogenic assay, for rapid radiosensitivity assessments of cultured cell lines and, potentially, of primary tumor cells obtained from biopsies. (3) To examine the strengths and feasibility of the assay in enabling time-efficient results regarding the evaluation of the potency of DDR inhibitors in radiosensitizing cells. Specifically, exponentially growing 82-6 hTERT human fibroblasts and human epithelial RPE cells are irradiated during the G2- to M-phase transition, and the contribution of ATR, ATM, and Chk1 inhibition to chromatid break yield is analyzed. Experiments were carried out with untreated cells, as well as with cells incubated with the ATM/ATR inhibitor Caffeine, the ATR inhibitor VE-821, and the CHK1 inhibitor UCN-1 to suppress the G2-checkpoint activation. As a result, the time for repair decreased and cells progressed to the metaphase with an increased yield of chromatid breaks. Analysis of chromatid breaks in the presence or absence of the DDR and G2-checkpoint inhibitors is a key modification in our G2-assay. In fact, the increased yield of chromatid breaks following treatment with the DDR inhibitors, which underpins cell radiosensitization to killing, is similar to that observed in the cells from highly radiosensitive AT-patients, and is considered as 100% radiosensitive internal control (26). Collectively, our observations highlight the potential use of the proposed modified G2-assay using VE-821 as an alternative to the conventional clonogenic assay for a time-efficient evaluation of cell radiosensitivity and the radiosensitizing efficacy of DDR inhibitors developed for genotoxic therapies.

## Materials and Methods

### Cell Culture and Irradiation Conditions

82-6 hTERT immortalized human fibroblasts were grown in the MEM medium with 10% fetal bovine serum (FBS) and antibiotics at 37°C in 5% CO2 and 95% air. Experiments were also carried out using retinal pigment epithelium (RPE) cells grown in the DMEM with 10% serum at 37°C in 5% CO2 and 95% air.

Irradiation was carried out using the X-ray machine (GE Healthcare) of the Institute of Medical Radiation Biology, University of Duisburg-Essen, Medical School, at room temperature. The machine was operated at 320 kV, 10 mA with a 1.65 mm Al filter (effective photon energy ~90 kV), at a distance of 50 cm, and a dose rate of ~1.3 Gy/min. Dosimetry was performed with a PTW or a chemical dosimeter. An even exposure to radiation of cell cultures was ensured by rotating the radiation table (27).

### Treatment of Cells With Kinase Inhibitors

Caffeine (Sigma-Aldrich) was dissolved in distilled water at 100 mM and used at a final concentration of 4 mM. 7-hydroxystaurosporine (UCN-01, Chk1i, Calbiochem) was dissolved in DMSO at 100 μM and was used at 50 nM final concentration. 3-Amino-6-[4-(methylsulfonyl)phenyl]-N-phenyl-2pyrazinecarboxamide (VE-821, ATRi, Haoyuan Chemexpress) was dissolved in DMSO at 10 mM and was used at a 2.5 μM final concentration, unless indicated otherwise. All inhibitors were added to exponentially growing cells 1 h before irradiation and were maintained until collection for analysis.

### The Modified G2-Assay–Statistical Analysis

For radiosensitivity testing using the classical G2-assay, the yields of chromatid breaks following the G2-phase irradiation of the test cells are compared to the distribution of the yields obtained for normal cells, e.g., lymphocytes obtained from a large number of healthy individuals. In our modified G2-assay, DDR inhibitors and G2-checkpoint abrogators are used to obtain the increased yields of chromatid breaks similar to those observed in cells of the highly radiosensitive AT patients (considered 100% radiosensitive). This increased yield of chromatid breaks in cells obtained from the same cell test system is employed here instead of the AT cells for radiosensitivity testing purposes, as a highly radiosensitive internal control (100% radiosensitive).

Specifically, the attached RPE and 82-6 hTERT exponentially growing cells were treated with three different DDR inhibitors: Caffeine (4 mM), VE-821 (2.5 μM), and UCN-01 (50 nM) 1 h before the irradiation with 0.5 or 1 Gy. The spontaneous aberration yield was subtracted to obtain the radiation-induced yield of chromatid breaks (CB), as well as the yield obtained in the presence of each inhibitor (CBinh). Using these two yields at 0.5 or 1 Gy with and without inhibitors, the following three “G2-assay” parameters were calculated as described below: Cell line resistance parameter, RP = CBinh-CB; G2-checkpoint effectiveness parameter, EP = (CBinh–CB)/CBinh; and the cell line individual radiosensitivity parameter, IRS = [1–(CBinh-CB)/CBinh] × 100%.

For the analysis of the radiation-induced chromatid breaks, metaphases were analyzed at 1 and 2 h post-irradiation. To accumulate the metaphases, colcemid was added for each time point during the final hour. Cells were collected by trypsinization and, following centrifugation, standard procedures were used for chromosome preparation and staining. Briefly, cells were treated in a hypotonic 75 mM KCl for 10 min and fixed two times in methanol:glacial acetic acid (3:1 v/v). Fixed cells were then spread on microscope slides, air-dried, stained in 3% Giemsa, and processed for cytogenetics analysis. For each experimental point, ~50 cells were scored for chromatid damage. Chromatid breaks and gaps were scored; the latter, only when it was longer than a chromatid width. For chromosome analysis, metaphases were located manually and analyzed using an image analysis system.

Standard deviations of the mean values from three independent experiments were calculated. Statistical testing was performed using a one-tailed Student's *t*-test, and a *p*-value of *p* ≤ 0.05 was considered of borderline statistical significance. Furthermore, a two-tailed Student's *t*-test was used to compare the difference between the three inhibitors, adopting the significance criterion of *p* ≤ 0.05.

## Results

**Figure 1:**
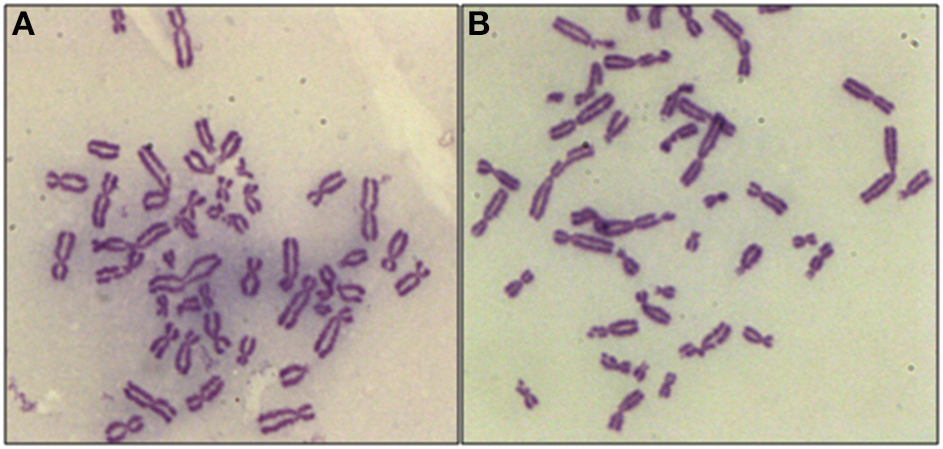
Representative examples of CBs in RPE metaphases treated with VE-821 or left untreated. **(A)** CBs in a cell analyzed 1 h after exposure to 1 Gy. **(B)** As in A for a cell also exposed to VE-821 1 h before IR. Note the increase in the yields of CBs.

The core hypothesis in this work is that the inhibition of radiation-induced G2-checkpoint via the inactivation of key regulated kinases will cause radiosensitization, and that this radiosensitization will be accompanied by an increase in the yields of CBs. We analyzed, therefore, the effects of Caffeine, VE-821, and UCN-01 on the CB yields in the irradiated 82-6 hTERT and RPE cells, using the G2-chromosomal radiosensitivity assay (26) with some modifications. When exponentially growing cells are irradiated and the metaphases are analyzed for CBs 1–2 h later, only the cells irradiated in the G2-phase are assayed and the response measured reflects that of the G_2_-phase-irradiated cells. Figure 1A shows a metaphase cell collected 1 h after exposure to 1 Gy. When cells are treated with VE-821 (Figure 1B), more chromatid breaks are scored. We conclude that abrogation of the G2-checkpoint through ATR inhibition compromises the processing of chromatid breaks.

**Figure 2:**
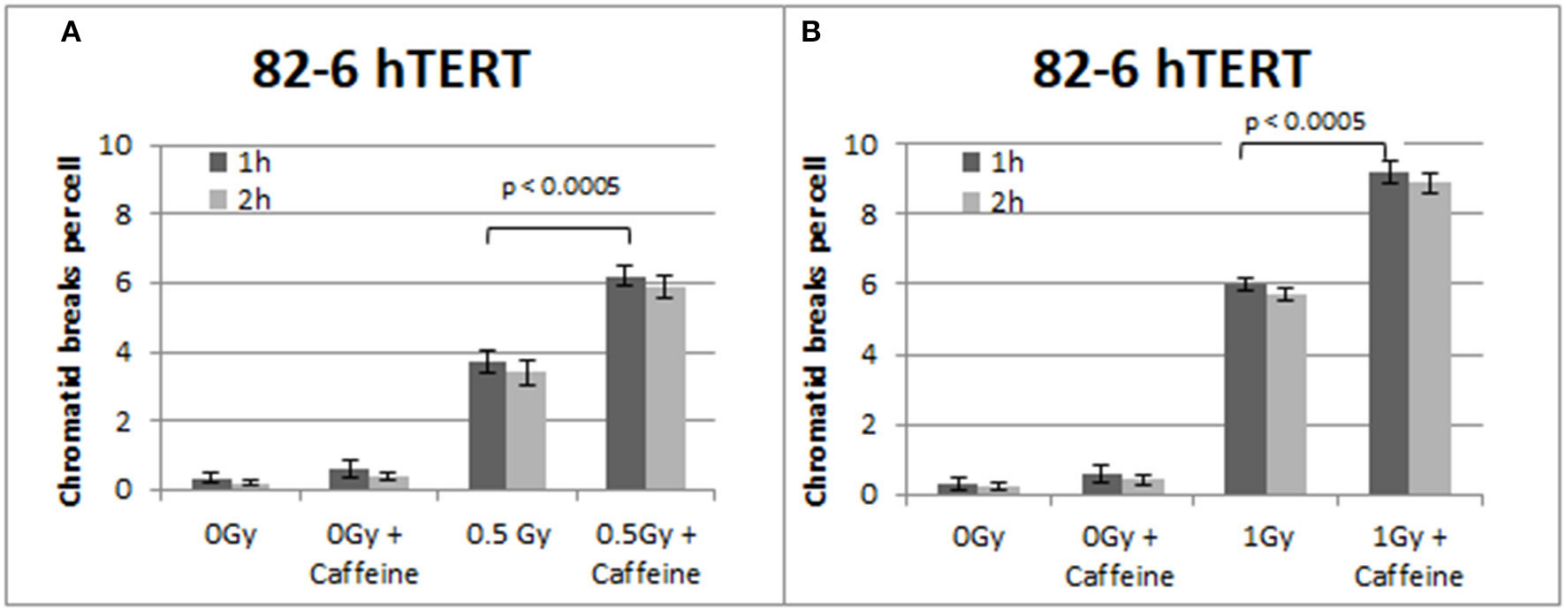
**(A)** Yield of CBs in 82-6 hTERT cells, treated with Caffeine or left untreated, following exposure to 0.5 Gy. **(B)** As in **(A)** for cells exposed to 1 Gy. The cells were harvested at 1 and 2 h after IR. Both graphs also show the yield of chromatid breaks in the unirradiated control groups (Mean ± SD based on three independent experiments; statistically significance criterion: *p* ≤ 0.05).

Figure 2 summarizes the results obtained with the 82-6 hTert cells exposed to 0.5 or 1.0 Gy and analyzed 1 or 2 h later. Cells were either left untreated or treated with Caffeine to abrogate the G2-checkpoint. The yields of CBs obtained at 1 h following exposure to 0.5 and 1 Gy was 3.7 and 6 CBs/metaphase, respectively. However, in the presence of Caffeine, the yields increased to 6.2 and 9.2 CBs/cell, respectively, following exposure to 0.5 or 1 Gy. There is also a small increase in the number of CBs/cell after the treatment with Caffeine in the sham-irradiated cells, but it fails to reach a statistical significance (*p* > 0.1). To optimize the G2-assay with reference to the harvesting times, mitotic cells were collected at two different post-irradiation time points–1 and 2 h. No significant differences in the CB numbers were observed between the two harvesting time points examined. We conclude that for the purpose of the G2-assay, the 1 h harvest time point typically used is sufficient.

**Figure 3:**
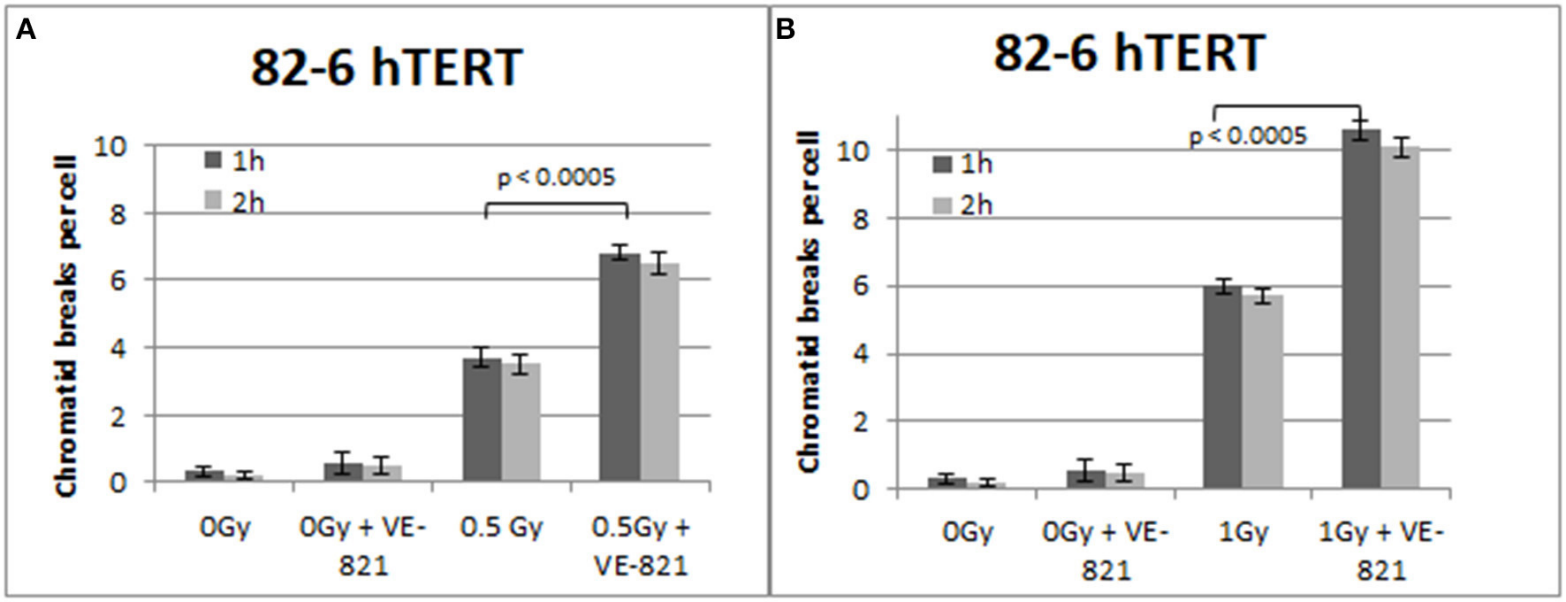
**(A)** Yield of chromatid breaks in the 82-6 hTERT cells, treated with VE-821 or left untreated, following exposure to 0.5 Gy. **(B)** As in **(A)** for cells exposed to 1 Gy. Other details as in Figure 2 (Mean ± SD based on three independent experiments; statistically significance criterion: *p* ≤ 0.05).

**Figure 4:**
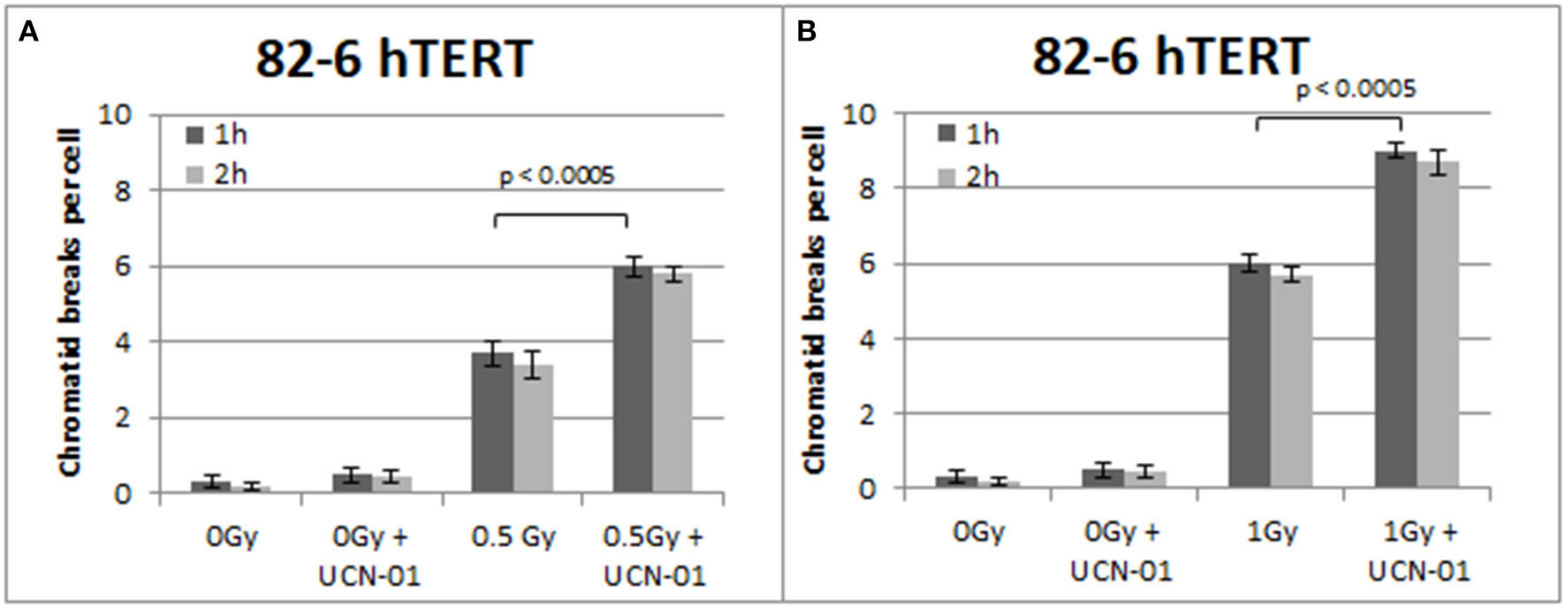
**(A)** Yield of chromatid breaks in the 82-6 hTERT cells, treated with UCN-01, or left untreated, following exposure to 0.5 Gy. **(B)** As in **(A)** after exposure to 1 Gy. Other details as in Figure 2 (Mean ± SD based on three independent experiments; statistically significance criterion: *p* ≤ 0.05).

Similar trends in the yields of chromatid breaks were also obtained for the 82-6 hTERT cells when the G2-checkpoint was abrogated using VE-821 (Figure 3) or UCN-01 (Figure 4). Specifically, the mean values of chromatid breaks were at 3.7 and 6 chromatid breaks/cell after exposure to 0.5 and 1 Gy, respectively (Figure 3). Interestingly, in the presence of VE-821, the yields increased to 6.8 and 10.6 chromatid breaks/cell following exposure to 0.5 or 1 Gy, respectively. A non-significant increase in the chromatid breaks after treatment with VE-821 was also observed in the sham-irradiated cells as compared in the control cells (*p* > 0.1). Furthermore, VE-821 significantly increased the number of CBs compared to Caffeine at 0.5 Gy and at 1 Gy (*p* < 0.01), as shown in **Figure 8**, and compared to UCN-01 (*p* < 0.01). However, no significant differences in the CB numbers were observed between Caffeine and UCN-01 at 0.5 Gy and at 1 Gy (*p* > 0.05).

Figure 4 shows the results obtained with the 82-6 hTert cells exposed to 0.5 or 1.0 Gy and analyzed 1 or 2 h later. Cells were either left untreated or treated with UCN-01 to abrogate the G2-checkpoint. The mean value of chromatid breaks 1 h after exposure to 0.5 and 1 Gy was 3.7 and 6 CBs/cell, respectively, and increased to 6 and 9 CBs/cell after treatment with UCN-01. There is also a very small increase in the number of CBs/cell after treatment with UCN-01 in the sham-irradiated cells as compared to the control cells (*p* > 0.1).

**Figure 5:**
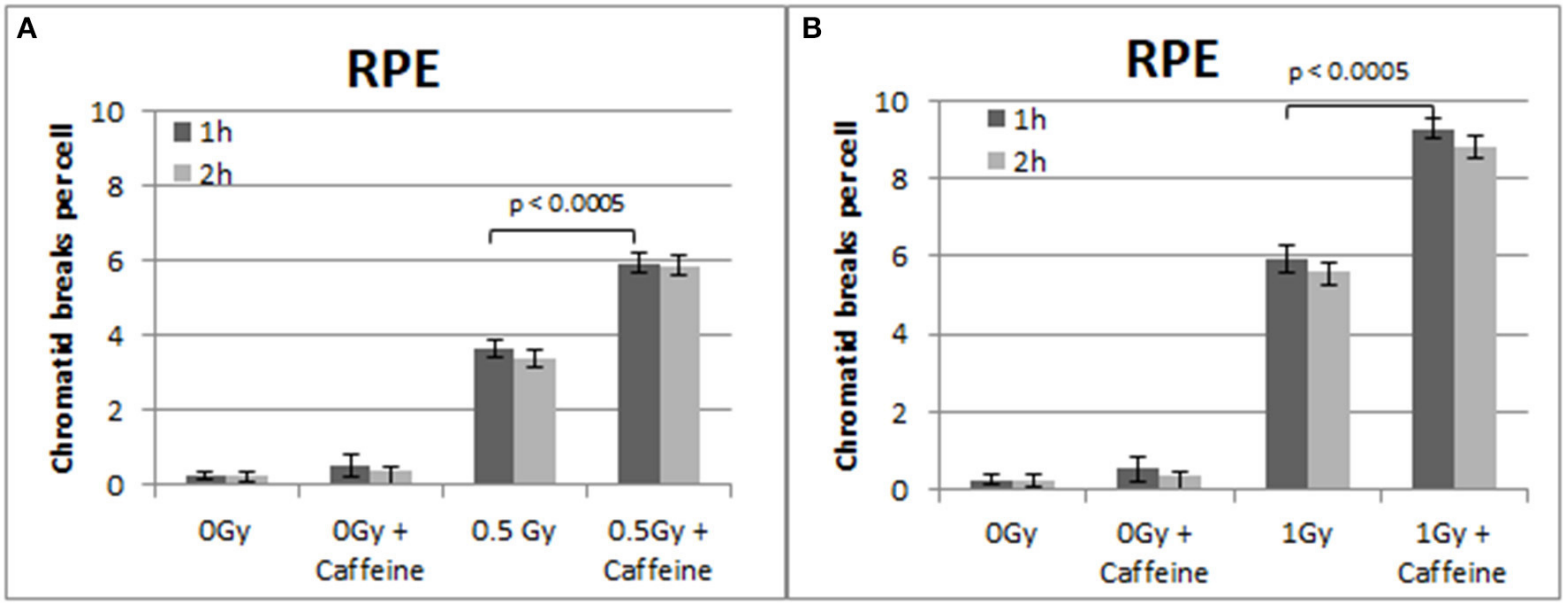
**(A)** Yield of chromatid breaks in the RPE cells, treated with Caffeine or left untreated, after exposure to 0.5 Gy. **(B)** As in **(A)** for cells exposed to 1 Gy. Other details as in Figure 2 (Mean ± SD based on three independent experiments; statistically significance criterion: *p* ≤ 0.05).

**Figure 6:**
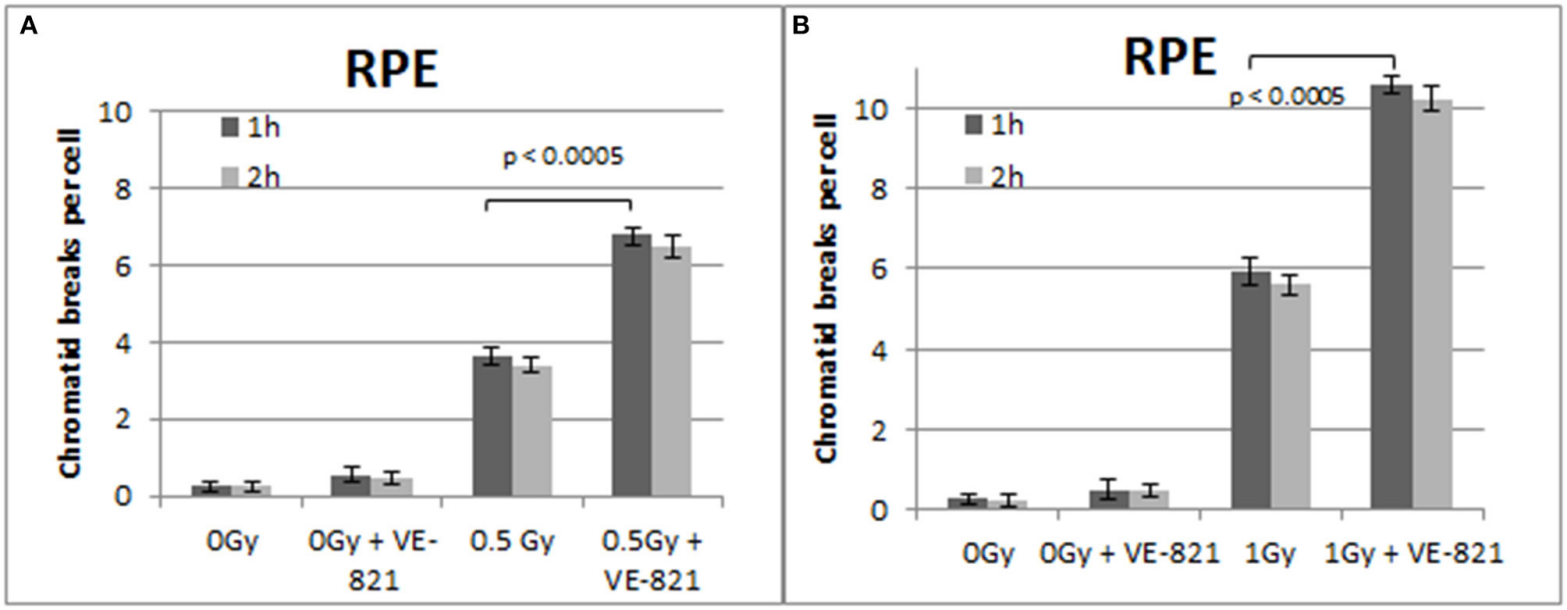
**(A)** Yield of CBs in the RPE cells, treated with VE-821 or left untreated, following exposure to 0.5 Gy. **(B)** As in **(A)** for cells exposed to 1 Gy. Cells were harvested at 1 and 2 h after IR (Mean ± SD based on three independent experiments; statistically significance criterion: *p* ≤ 0.05).

**Figure 7:**
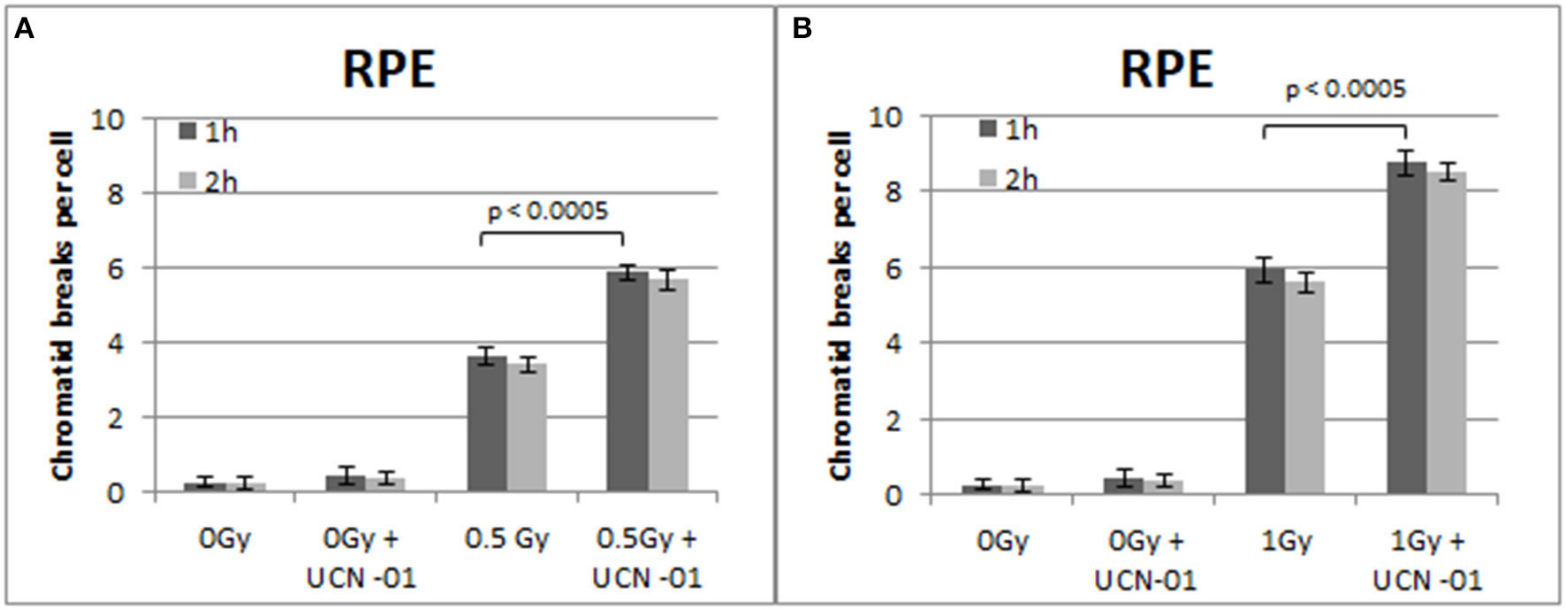
**(A)** Yield of chromatid breaks in the RPE cells, treated with UCN-01 or left untreated, following exposure to 0.5 Gy. **(B)** As in **(A)** for cells exposed to 1 Gy. Other details as in Figure 2 (Mean ± SD based on three independent experiments; statistically significance criterion: *p* ≤ 0.05).

To confirm that the above responses are not a peculiarity of the 82-6 hTert cells, we carried out similar experiments using the RPE cells. The results obtained are shown in Figures 5–7. Figure 5 shows that abrogation of the G2-checkpoint in the RPE cells treated with Caffeine increases the yield of CBs. Thus, following exposure to 0.5 and 1 Gy, CBs increased to 5.9 and 9.3 CBs/cell after treatment with Caffeine 1 h later (Figures 5A,B). We also observed a non-significant increase in chromatid breaks after treatment with Caffeine in the sham-irradiated cells (*p* > 0.1).

**Figure 8:**
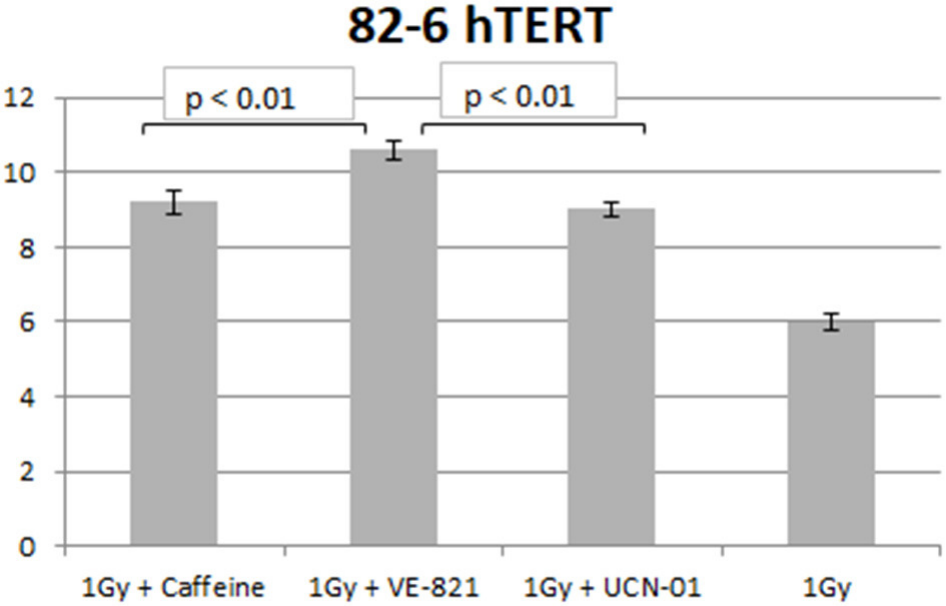
Yield of chromatid breaks in the 82-6 hTERT cells pre-treated with Caffeine, VE-821, UCN-01 or left untreated following exposure to 1 Gy (Mean ± SD based on three independent experiments; statistically significance criterion: *p* ≤ 0.05). VE-821 significantly increased the number of CB compared to Caffeine or UCN-01 (*p* < 0.01).

Similar trends in the yields of CBs were also obtained with the RPE cells when the G2-checkpoint was abrogated using VE-821 (Figure 6) or UCN-01 (Figure 7). Specifically, the mean number of CBs in the RPE cells 1 h after exposure to 0.5 Gy is 3.8 CBs/cell, while the mean number of chromatid breaks after treatment with VE-821 is 6.8 breaks/cell (Figure 6A). Following 1 Gy exposure, the yield of 6 CBs/cell increases to 10.6 CBs/cell after treatment with VE-821 (Figure 6B). There is a downward trend in the number of CBs 2 h later in the groups treated with VE-821 or left untreated (Figure 6). Furthermore, VE-821 significantly increased the number of CBs compared to Caffeine at 0.5 Gy and at 1 Gy (*p* < 0.01), as shown in Figure 8, and compared to UCN-01 (*p* < 0.01). However, there is no significant difference between Caffeine and UCN-01 at 0.5 or 1 Gy (*p* > 0.05).

Figure 7 shows the increased number of CBs in the RPE cells treated with the Chk1 inhibitor UCN-01 after exposure to 0.5 and 1 Gy. The yields of CBs obtained 1 h following exposure to 0.5 and 1 Gy is 3.6 and 5.8 CBs/cell, respectively. In the presence of UCN-01, the yields increase to 5.9 and 8.7 CBs/cell following exposure to 0.5 or 1 Gy. Figure 7 also shows a small non-significant decrease in the number of chromatid breaks 2 h after exposure in all groups.

Table 1 presents the calculated values of the G2-assay parameters, defined under the Materials and Methods, using the 82-6 hTERT and RPE cells exposed to the indicated IR doses and analyzed at the indicated post IR times, when either left untreated or incubated with the indicated inhibitors. The first parameter RP, which is related to the resistance of the cell line to radiation and to the potency of an inhibitor to abrogate the radiation-induced G2/M checkpoint, is defined as the difference CBinh-CB. Higher RP values are observed when the VE-821 ATR inhibitor is used in both cell lines after exposure to 1 Gy. The second parameter is the effectiveness parameter of the G2 checkpoint (EP) as revealed by the inhibitor used and calculated according to the formula (CBinh-CB)/CBinh). The highest EP values are observed with VE-821 at 0.5 and 1 Gy. Based on the effectiveness parameter of the G2-checkpoint, the individual radiosensitivity is evaluated by the IRS = [1–(CBinh–CB)/CBinh] × 100%, i.e., as percentage of the highly radiosensitive AT cells used as an internal control (100% radiosensitive) (26). The values of IRS parameters for the 82-6 hTERT and RPE cell lines treated with Caffeine, VE-821, and UCN-01 are also presented in Table 1.

**Table 1:** G2-assay parameters (1 h post-irradiation with 0.5 and 1 Gy).

**Cell line**	**82-6 hTERT**						**RPE**					
**Dose**	**0.5 Gy**			**1.0 Gy**			**0.5 Gy**			**1.0 Gy**		
**Inhibitors**	**Caf**.	**VE-821**	**UCN-01**	**Caf**.	**VE-821**	**UCN-01**	**Caf**.	**VE-821**	**UCN-01**	**Caf**.	**VE-821**	**UCN-01**
RP (breaks/cell)	2.5	3.1	2.3	3.2	4.6	3.0	2.3	3.1	2.3	3.4	4.7	2.9
EP	0.4	0.45	0.38	0.35	0.44	0.33	0.38	0.45	0.39	0.37	0.44	0.33
IRS(% of AT cells radiosensitivity)	60%	55%	62%	65%	56%	67%	61%	54%	62%	63%	55%	67%

## Discussion

When actively growing populations of cells are exposed to IR and metaphases are analyzed for cytogenetic damage 1 h later, chromatid breaks are exclusively assessed. This represents the response of cells that were in the last part of the G_2_-phase at the time of irradiation. The approach and the related protocol are frequently referred to as the “G_2_-assay” to reflect the phase of the cell cycle in which the induced DNA damage, if not repaired, is transformed into chromatid breaks as cells proceed to the metaphase. High relative numbers of chromatid breaks (CB) detected by the “G_2_-assay” have been proposed to be predictive of cancer propensity (28) and cell radiosensitivity to killing (26, 29–32).

When the assay includes the measurements of chromatid breaks at the metaphase at multiple time points after IR, instead of only the 1 h time point, it detects repair of a subset of DSBs that have the distinct property of breaking the chromosomes—estimated to be ~10% of the total DSBs induced. This approach has been extensively used by Peter Bryant to study the pathway engagement in the repair of this specific subset of DSBs in the G_2_-phase (33). We have extensively used the same approach to study the pathway engagement for this subset of DSBs, and our recent work demonstrates that at low doses of IR (<2 Gy), repair of chromatid breaks requires active HR (27) and intact checkpoints (34), suggesting an epistatic relationship between these two endpoints. Notably, as the IR dose increases, contributions by c-NHEJ become clearly detectable in an impressive demonstration of a dose-dependent pathway switch from HR to c-NHEJ (35).

The results presented here show that when the exponentially growing RPE and 82-6 hTERT cells are analyzed using our modified protocol of the G_2_-assay, including suppression of the G2-checkpoint response using Caffeine, VE-821, or UCN-01, a marked increase of CB yields are obtained. This result is in line with the above indicated requirement for intact checkpoints for an efficient CB repair that mainly relies on HR. We postulate that the increase in the CB measured after treatment with the checkpoint inhibitors reflect the repair inhibition of essential subsets of DSBs, which are converted into chromatid breaks as G2-cells are forced to enter the M-phase. This is well-known and documented by the dramatic increase of mitotic index and aberrant metaphases, which are subsequently reflected in cell radiosensitivity to killing. Therefore, the mechanistic insights underlying the effects of pretreatments with the inhibitors of DDR signaling in the G2-phase are mainly focused on their ability to disrupt the G2/M-checkpoint, thus, affecting DNA repair by decreasing the time available for repair of the radiation-induced DNA damage.

Chromosomal aberrations are the culprits of ionizing radiation-induced cell killing. For cells irradiated in the G_2_-phase of the cell cycle, all known DSB repair pathways (HR, c-NHEJ, alt-EJ, and SSA) are active and, in principle, capable of processing DSBs. These processing benefits from the activation of the G2/M checkpoint, which, by delaying cell progression into mitosis, gives the cell time to engage in DSB repair. Chromatin condensation associated with mitotic entry is likely to take apart the ends of unrepaired DSBs and make subsequent repair during the M-phase of the following G_1_-phase more difficult; it may also cause a mitotic catastrophe. Indeed, we have previously reported (31) that chromatin condensation during the G_2_ to M-phase transition facilitates the conversion of unrepaired DSBs into CBs. Not surprisingly, the G_2_-checkpoint is one of the strongest checkpoints activated by DSBs throughout the cell cycle (5). Thus, DSB repair mechanisms and cell-cycle regulation by checkpoint activation are important determinants of the G2-phase cell radiosensitivity (28), and our recent demonstration for the epistatic requirement of HR and checkpoints to achieve CB repair in the G_2_-phase is in line with this expectation (27, 34). In this intellectual background, therapeutic radiosensitivity could be improved equally efficiently either by checkpoint abrogation or by suppression of HR (34). The results presented here corroborate this expectation. Our results are in line with the documented role of ATM and ATR kinases in the G_2_-checkpoint activation, and suggest that the response of the 82-6 hTert fibroblasts is similar to that of the epithelial RPE cells. Caffeine, by inhibiting ATM and ATR, causes an ~60% increase in CB, while VE-821, by highly specifically inhibiting ATR, causes an almost 80% increase in CB. UCN-01, on the other hand, increases CB by only ~50%. These responses are in line with our previous results on the mechanistic regulation of the G_2_-checkpoint in cells irradiated in the G_2_-phase of the cell cycle (13). Furthermore, VE-821 significantly increased the number of CB compared to Caffeine and UCN-01 at 0.5 and 1 Gy, as shown in Figure 8, for both cell lines used (*p* < 0.01).

The G_2_-assay, as modified here and as outlined before (26, 31, 32, 36, 37), can be very useful in the determination of intrinsic radiosensitivity to killing of a particular cell line, and may also be used to quantitate radiosensitization following treatment with DDR inhibitors. Particularly, the difference CBinh-CB (in the yields of chromatid breaks in the presence or absence of an inhibitor, respectively) is related to the resistance parameter (RP) of the cell line to radiation, and to the potency of a selective inhibitor to abrogate the radiation-induced G2/M checkpoint. The higher the RP value, the higher the resistance of the cell line in the G2 phase, and the potency of a selective inhibitor to abrogate the G2-checkpoint arrest. In contrast, the lower the value of this parameter, the higher the radiosensitivity of the cell line used. As the RP value approaches zero, the radiosensitivity of the cell line will be close to that of the highly radiosensitive Ataxia Telangiectasia (AT) cells, which have compromised the G2/M checkpoint (31).

Furthermore, the effectiveness parameter of the G2 checkpoint (EP), calculated as the ratio (CBinh-CB)/CBinh, reflects the potency of DDR to resolve the radiation-induced DNA damage (37), and revealed by the use of the DDR inhibitors. Therefore, the higher the EP value, the higher the resistance of a particular cell line to an ionizing radiation. In highly radioresistant cells (i.e., CB = 0), the EP value becomes 1, and in highly radiosensitive (i.e., CB = CBinh), it becomes 0. Based on the effectiveness parameter of the G2-checkpoint, the individual radiosensitivity (IRS) of a cell line can then be evaluated using the parameter IRS = [1–(CBinh–CB)/CBinh] × 100%, i.e., as a percentage of the high radiosensitivity level of cells from AT patients (100% radiosensitive) (26). Based on the G2-assay parameters and the RP values presented in Table 1, the inhibitor VE-821 exhibits the maximum potency to suppress CB repair, and has also been shown to effectively suppress the G2 checkpoint in the 82-6 hTERT cells, as well as in the RPE cells (13). Also, considering the EP values in Table 1, it can be concluded that the inhibitor VE-821 has the maximum potency in both cell lines used. Regarding the radiosensitivity testing by means of the G2-assay for the two cell lines used, the three inhibitors showed a radiosensitivity level from 55 to 67% for the 82-6 hTERT cells, and 54–67% for RPE cells, as compared to the highly radiosensitive cells of AT patients.

The use of the proposed modified G2-assay instead of the conventional clonogenic assay, predicts that both cell lines are equally radiosensitive. This conclusion agrees with the results that were recently reported by Soni et al. (38), and supports our hypothesis that an increased yield of chromatid breaks underpins radiosensitization to killing. Based on the G2-assay for the prediction of individual radiosensitivity reported by Pantelias and Terzoudi (26), normal human cells are considered to have up to 50% the radiosensitivity of AT cells. Consequently, when this value is compared to the IRS values revealed by the three inhibitors used in this work (Table 1), the potent G2-checkpoint inhibitor VE-821 gives the best prediction for the radiosensitivity of the RPE and 82-6 hTERT normal human cells (54–56% of AT radiosensitivity). The mechanism underpinning the high efficacy of VE-821 may be derived from its potential to abrogate very efficiently the G2-checkpoint, as reported recently by Mladenov et al. (13). These authors reported that ATR completely controls the G2 checkpoint induced in G2-phase cells exposed to low radiation doses. This is an unexpected and novel observation, as it is widely accepted that ATM is the main regulator of the G2-checkpoint, and that ATR has a much smaller role (13).

Therefore, our modified G2-assay using VE-821 instead of caffeine can be used as an alternative to the laborious conventional clonogenic assay. In fact, for both assays, basic expertise in tissue culture methods is required to obtain exponentially growing cells. From this stage on, however, the radiosensitivity testing using the G2-assay can be performed within 24 h, whereas more than 2 weeks are needed for the clonogenic assay. Of course, the G2-assay requires skilled cytogeneticists with experience in the analysis of chromatid breaks at metaphase cells. However, the experimentations can be considerably assisted currently by the availability of powerful image analysis systems, e.g., IKAROS software, MetaSystems, Germany.

Overall, the results generated here led to the development and optimization of a modified G2-assay using the VE-821 ATR inhibitor enabling time-efficient radiosensitivity assessments of cultured cells, and, potentially, of primary tumor cells obtained from biopsies. In fact, our observations paved the way to the testing of various cell lines using the G2-assay as an alternative of the conventional clonogenic assay. The assay could also be used as a powerful tool for radiosensitization screening of future DDR inhibitors. Such inhibitors are developed with the idea to sensitize cancer cells to DNA damaging agents without inducing severe systemic toxicities in normal tissues. Furthermore, our results using radiation cytogenetics reinforce the concept that ATM, ATR, and Chk1 represent attractive anticancer drug targets in radiation oncology since (i) resistance to genotoxic therapies has been associated with an increased DDR signaling, and (ii) many cancers have defects in certain components of the DDR, rendering them highly dependent on the remaining DDR pathways for survival.

## Data Availability Statement

The raw data supporting the conclusions of this article will be made available by the authors, without undue reservation.

## Author Contributions

AN: conceptualization, investigation, analysis, and writing—original draft preparation. AS: supervision of experimental work and review of the manuscript. MH: aided in the analysis. PK: review of manuscript. GP: critical reading and editing of the manuscript. GT: supervision, data interpretation, scientific guidance, and critical review of the manuscript. GI: supervision, data interpretation, scientific guidance, and revision of the manuscript. All authors contributed to the article and approved the submitted version.

## Conflict of Interest

The authors declare that the research was conducted in the absence of any commercial or financial relationships that could be construed as a potential conflict of interest.
